# Characterization of the Different Subtypes of Immune Cell Infiltration to Aid Immunotherapy

**DOI:** 10.3389/fcell.2021.758479

**Published:** 2022-03-15

**Authors:** Zhenqing Li, Kai Mao, Bo Ding, Qun Xue

**Affiliations:** ^1^ Cardiovascular Surgery Department, Affiliated Hospital of Nantong University, Nantong, China; ^2^ Medical College of Nantong University, Nantong, China; ^3^ Research Center of Clinical Medicine, Affiliated Hospital of Nantong University, Nantong, China

**Keywords:** lung adenocarcinoma, PD-1 inhibitor, immune cell infiltration, TCGA, PCA

## Abstract

**Background?**PD-1 ablation or PD-L1 specific monoclonal antibody against PD-1 can recruit the accumulation of functional T cells, leading to tumor rejection in the microenvironment and significantly improving the prognosis of various cancers. Despite these unprecedented clinical successes, intervention remission rates remain low after treatment, rarely exceeding 40%. The observation of PD-1/L1 blocking in patients is undoubtedly multifactorial, but the infiltrating degree of CD8+T cell may be an important factor for immunotherapeutic resistance.

**Methods:**We proposed two computational algorithms to reveal the immune cell infiltration (ICI) landscape of 1646 lung adenocarcinoma patients. Three immune cell infiltration patterns were defined and the relative ICI scoring depended on principal-component analysis.

**Results:**A high ICI score was associated with the increased tumor mutation burden and cell proliferation-related signaling pathways. Different cellular signaling pathways were observed in low ICI score subtypes, indicating active cell proliferation, and may be associated with poor prognosis.

**Conclusion:**Our research identified that the ICI scores worked as an effective immunotherapy index, which may provide promising therapeutic strategies on immune therapeutics for lung adenocarcinoma.

## Introduction

Lung cancer is a leading cause of death, resulting in the deaths of around 1.6 million people per year (Bray et al., 2018). On the basis of histological types of tumor tissues, lung cancer can be classified as small cell lung cancer (SLC) and non-small cell lung cancer (NSCLC); NSCLC accounts for about 87% of total lung cancer patients (DeSantis et al., 2014). Among these lung cancer types, the.

5-years overall survival rate of NSCLS is only about 27% and most NSCLC is diagnosed at the advanced stage, which also contributes to the poor survival rate (Luo et al., 2019). To extend the overall survival of NSCLC patients, many advanced diagnosis and treatment methods have been introduced in clinical interventions, including targeted molecular therapy (Qian and Massion, 2018; Wu et al., 2020a) and immunotherapy (Corrales et al., 2018; Hao et al., 2020). To precisely provide therapeutic interventions, precise molecular classification and diagnosis can significantly improve the overall survival of NSCLC patients, for example osimertinib for EGFR T790M patient therapy (Mok et al., 2017).

Recently, many investigations have proved that immune evasion is deeply affected by immune therapy (Topper et al., 2017; Takahashi et al., 2019). However, expansive landscapes of immune cell infiltration and the microenvironment (TME) of LUAD has not been well investigated. Thus, it is crucial to identify the roles of various TME in LUAD to conduct individual immune therapies. The over-activated inflammatory pathways play a crucial role in the tumorigenesis, which evolve into various cancer-related oncogenic pathways (Hanahan and Weinberg, 2011). These heterogeneities of cancers lead to the failure of therapy, and it is suggested that individual immunotherapy strategies may be helpful to improve the overall survival of cancer patients. In the tumorigenesis stage, the immune surveillance mechanisms have been well validated, and indicated that immunotherapies can lead to an improved immune response for more successful anticancer therapy (Mohme et al., 2017). To perform these therapeutic strategies, various types of immunotherapeutic approaches have been developed, for example vaccine therapy (Vermaelen, 2019), chimeric antigen receptor (CAR) T cells (Zeltsman et al., 2017), programmed cell death 1 (PD-1), and programmed cell death ligand-1 (PD-L1) (Freeman et al., 2000; Dong et al., 2002; Curiel et al., 2003).

Despite these unprecedented clinical successes in anticancer treatment, the immunotherapy remission rates of various types of cancers still remain low. Meanwhile, immune tolerance is obtained after receiving immunotherapy, due to cross-talks between tumorigenesis and immune-response (Munn and Mellor, 2016). In recent decades, next-generation sequencing (NGS) has been promoted in the development of biomedical field and obtained great achievements (Gu et al., 2019). Moreover, the NGS algorithm technology also revealed large amounts of biological information about cancer tissue (Brandsma et al., 2019). To identify the percentage of immune cell infiltration, two algorithms have been used: CIBERSORT (Newman et al., 2015) and ESTIMATE (Yoshihara et al., 2013). By independently utilizing these algorithms, different combinations of biomarkers can be well-related for the prognosis of cancer patients (Yoshihara et al., 2013; Min et al., 2019).

In this research, we utilized both CIBERSORT and ESTIMATE to analyze the gene expression information of tumor tissues, including the immune cell infiltration and spatial distribution, or the so-called intra-tumoral immune landscape. Here, we identified that the LUAD can be divided into three subtypes based on the immune cell-infiltration patterns. Finally, we conducted a correlation analysis between ICI score and PD-1/PD-L1 immunotherapy response or various immune landscapes, which indicated that ICI score could be used as the clinical prediction index for LUAD patients to receive immunotherapy.

## Materials and Methods

### Datasets and Samples

A total of 1646 patients’ mRNA expressions with detailed survival information were downloaded from TCGA-LUAD from TCGA database (https://portal.gdc.cancer.gov/)and GEO datasets including GSE31210, GSE30219, GSE68465, GSE72094, TCGA-LUAD, GSE30219, and GSE72094.

### Data Normalization

After merging multiple data sets across platforms, the normalization of data was acquired. Expression values were log-transformed and we used the “ComBat” algorithm to reduce the possibility of batch effects due to non-biotech bias between different data sets (Johnson et al., 2007).

### Consensus Clustering for Tumor-Infiltrating Immune Cells

The gene expression matrix data was uploaded to the CIBERSORT by employing the LM22 signature and 100 permutations were set to obtain the immune cell infiltration matrix (Newman et al., 2015). Meanwhile we used the ESTIMATE package to evaluate the immune and stromal score per LUAD sample (Yoshihara et al., 2013). These samples were stratified and clustered based on the ICI pattern. In the analysis, we used the “hc” method of the unsupervised clustering with Pearson, and both innerLinkage and finalLinkage were set to Ward. D2, executed by using the “ConsensusClusterPlus” R package (Yu et al., 2012). In order to make the classification steady, the maxK was set to three and the reps was set to 100 times.

### Differential Expression Genes Associated With the Immune Cell Infiltration Phenotype

Based on the ConsensusClusterPlus analysis, these patients were grouped into three ICI groups. Meanwhile, we used the limma package to identify the differential expression genes (DEGs) in those ICI clusters with significance cutoff criteria *p* < 0.05 (adjusted) and absolute fold-change > 1.5.

### Dimension Reduction and Generation of Immune Cell Infiltration Score

Basing on the three ICI clusters, we used consensus clustering (parameters: reps:100, pItem:1, pFeature:1; Ward. D2 and euclidean distance, k:4) using expression values of those DEGs to identify the gene clusters; meanwhile, in order to reduce the relevant variables and unnecessary noise, we used the Boruta package to reduce dimension. When the DGEs were positive the cluster was marked as signature A and when it was negative it was marked signature B. Then we identified the principal component 1 as the signature score by using the PCA analysis. Finally, we constructed an approach that is similar to the gene expression grade index to identify the ICI score of various patients: ICI score = PC1A- PC1B:

### Collection of Somatic Alteration Data

To confirm the tumor mutational burden of lung adenocarcinoma, we download the mutation data from cBioPortal (https://www.cbioportal.org/; luad_tcga_pan_can_atlas 2018) and evaluated the entire number of non-synonymous mutations in LUAD. In both the high ICI score and low ICI score groups, we used the “maftool” R package to analyze the somatic alterations, especially the top 25 driver genes with the highest alteration frequency in lung adenocarcinoma.

### Gene Expression Data With Immunotherapy

For further immunotherapy research, we download the IMvigor210 dataset to analyze the value of ICI scores in the PD-1 response predicted, which can be acquired from http://research-pub.gene.com/IMvigor210CoreBiologies with completed information about the response to PD-L1 blockade.

### Analysis of the Validation Set

We downloaded a data set including 535 lung adenocarcinoma patients from TCGA database (https://portal.gdc.cancer.gov/cart), and then used TIDE website (Jiang et al., 2018) to predict the cancer immunotherapy response for the 535 patients. The 535 lung adenocarcinoma patients were then divided into two groups according to the TIDE prediction: Response group and Non-response group. Finally, we performed the ICI score analysis for these two groups and compared the results with the TIDE results.

### Statistical Analyses

We utilized Graphpad prism and R 4.0.0 software to conduct all statistical analyses. The Kruskal–Wallis test and Wilcoxon test was used to compare two groups and more than two groups. In each dataset, survival curves for subgroups were analyzed by using the Kaplan-Meier plotters. Chi-square test was used to analyze the correlation between ICI score subgroup and somatic mutation frequency, and Spearman analysis was used to calculate the correlation coefficient. *p* < 0.05 was considered as statistically significant.

## Result

### The Landscape of Immuno-Cell Infiltration in the The Microenvironment of LUAD

Firstly, we used the “ComBat” algorithm to reduce the possibility of batch effects due to non-biotech bias between different data sets (Supplementary Figures S1A, B). Then, the CIBERSORT and ESTIMATE algorithms were employed to identify the immuno-cell infiltration in LUAD tumor tissues. Based on 1646 tumor samples with matched immune cell infiltration (ICI) profiles from the meta-cohort (Array express database: GSE31210, GSE30219, GSE68465 and GSE72094; The *Cancer* Genome Atlas TCGA-LUAD), unsupervised clustering of LUAD patients into different subtypes was performed as described in the MATERIAL and METHOD section.

**FIGURE 1:**
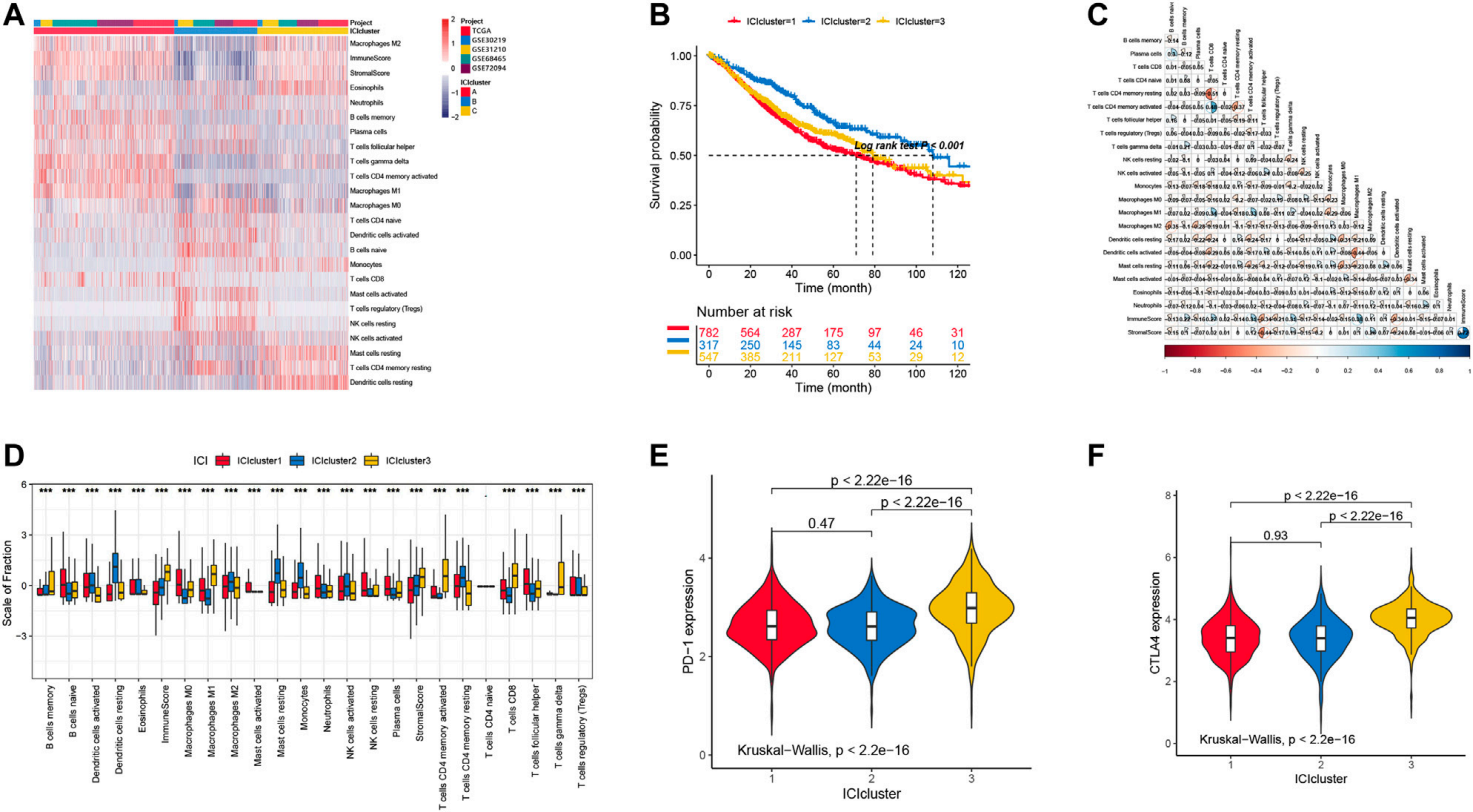
**(A)** The landscape immune cell infiltration in unsupervised clustering analysis with 1646 samples from the data sets described in the methods. **(B)** Survival curves in three clusters of LUAD patients with different immune cell infiltration classes (*p* < 0.001). **(C)** The correlation in different immune cells in the tumor patients. **(D)** The distribution of tumor-infiltrating immune cells in three ICI clusters. **(E)** PD-1 expression level of immune cells in different ICI clusters. **(F)** CTLA4 expression level of immune cells in different ICI clusters.

To our knowledge, the immune cell infiltration condition is highly associated with the patients’ prognosis. To identify the role of specific immune cells in tumor progression, classification of LUAD based on the immune cell infiltration was employed. Here, three ICI clusters were identified with various infiltrated immune cells (Figure 1A). We also found that the ICI cluster 2 has a favorable prognosis (Figure 1B). Considering the immune landscape of LUAD (Figure 1A), the infiltration percentage of Dendritic cells resting, Dendritic cells activated, Macrophages M2, Mast cells resting, NK cells activated, and T cells CD4 memory resting in the ICI cluster 2 were significantly higher than ICI cluster 1 and 3.

The landscape of immune cell interaction in TME are pictured in Figure 1C. We can observe that the correlation between T CD8^+^ cell and T CD4^+^ cell had more memory resting and activated cells than other infiltration immune cells. Moreover, we also tested the PD-1 and CLAT4 expression level in each ICI cluster by using the Kruskal–Wallis test, and the results showed that cluster 3 has higher expression levels with significant difference (Figures 1D,F). However, we cannot observe any difference between cluster 1 and 2, in which the expression level of PD-1 and CTLA4 is similar.

### Identified Immune Gene Subtype

To confirm the underlying biological functions in different immune-phenotypes, we employed the Limma R package to identify the differential expression genes. Because the clinical information of the TCGA-LUAD cohort is not complete, we mainly used LUAD patients with entire clinical information to further perform the analysis in the following study.

**FIGURE 2:**
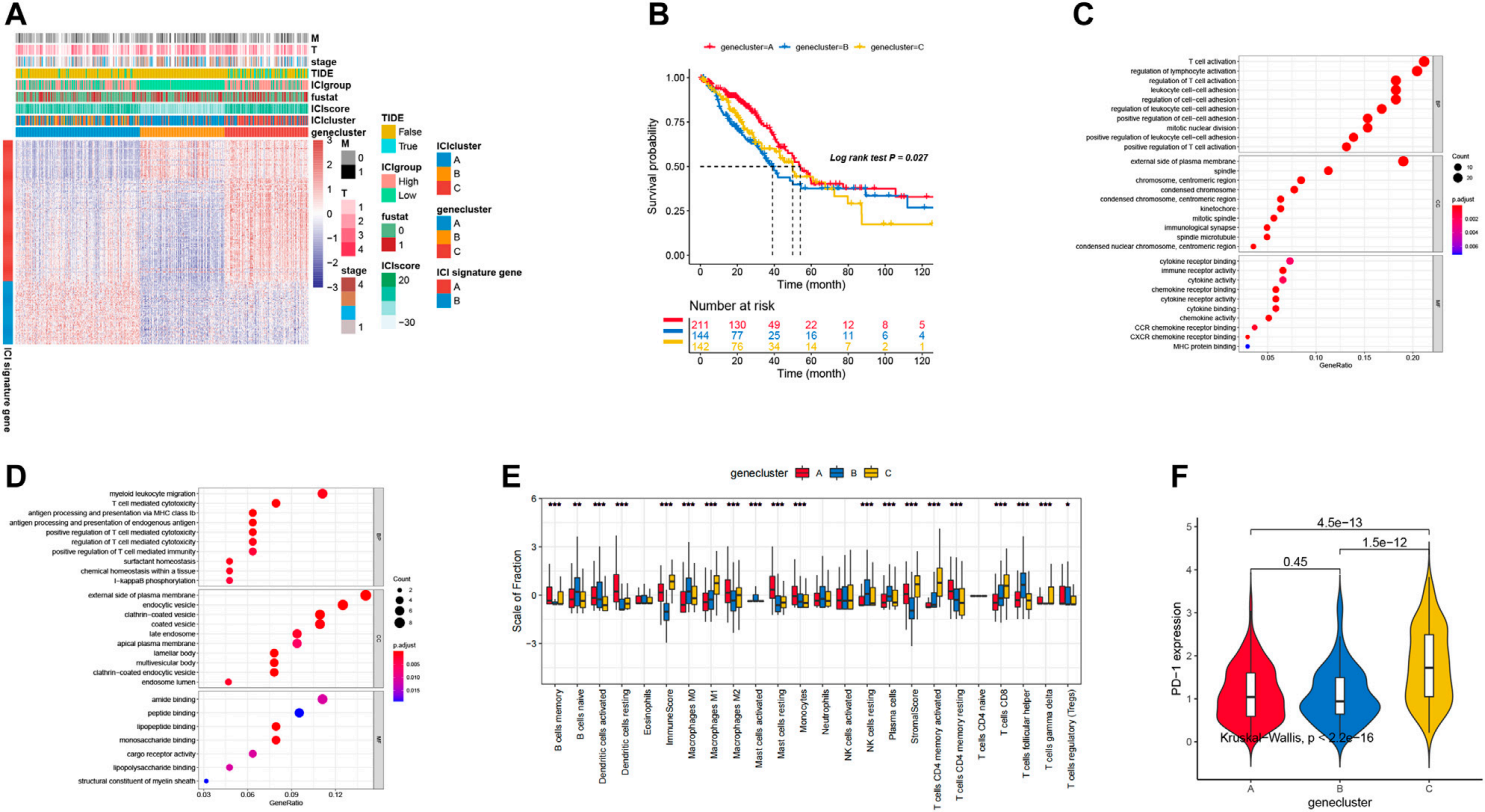
**(A)** Unsupervised clustering analysis in DEGs of three ICI clusters into three groups: gene clusters A, B, and C. 535 patients in the TCGA-LUAD cohort with complete clinical information were included into the analysis. **(B)** Survival curves for three gene clusters. **(C,D)** The biological function of two signature clusters: signature A and B. **(E)** The distribution of immune cell infiltration in three gene clusters. **(F)** PD-1 expression level in different gene clusters.

Firstly, we analyzed the aforementioned differentially expressed genes (DEGs) by using the ConsensusClusterPlus package with the unsupervised clustering analysis functions, and these genes can be classified into three genomic clusters: gene clusters A, B, and C. To reduce the noises of cluster genes, we used the Boruta package to reduce the dimension in both signature A and B. Here, we identified 144 genes, which were positively correlated with the gene cluster A. The remaining 64 genes were identified as signature B. All the gene expression in LUAD patients were pictured in Figure 2A, mainly based on the gene cluster types. To analyze by the expression level, gene cluster A is significantly different from the other two types, in which ICI signature gene A is lower and ICI signature B is higher. These results are consistent with the prognosis of LUAD patients based on the gene cluster classification (Figure 2B).

Moreover, we also performed the Gene Ontology (GO) analysis to identify the role of differentially expressed genes. As shown in Figures 2C,D, some signal pathways associated with T regulation processes and cytokines secretion were enriched, for example positive regulation of T cell activation and T cell activation. These results indicated that the T cell regulation related pathways played a critical role in promoting the tumorigenesis.

Previous investigations have proven that the immune system may display different roles with both favorable and adverse outcomes, which is determined by the role of immune cells as either pro-tumor or anti-tumor, as we observed in our previous analysis. As shown in Figure 2E, gene cluster A exhibited higher B cells memory and more Dendritic cells activated, Dendritic cells resting, Macrophages M2, Mast cells resting, Monocytes, and T cells CD4 memory resting. These immune cells displayed the highest level of infiltration, and may evolve into improving the therapy. PD-1 is the critical biomarker for immunotherapeutic intervention and is always expressed on activated T, natural killer and B lymphocytes, macrophages, dendritic cells, and monocytes (Ahmadzadeh et al., 2009). In addition, the expression level of PD-1 was significantly different among the three genomic clusters (Kruskal–Wallis, *p* < 0.01; Figure 2F). ICI gene clusters of A and B were associated with lower PD1/PD-1 expression levels, while ICI gene clusters C were associated with higher PD1/PD-L1 expression levels. Our investigation showed that the consistency of the immune profiles of different gene groups indicated the different prognostic profile.

### Construction of the Immune Cell Infiltration Score

**FIGURE 3:**
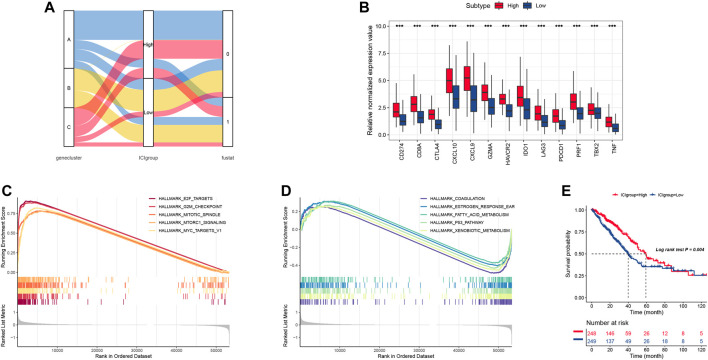
**(A)** The alluvial plot of ICI gene cluster distribution, ICI score, and survival outcome in different ICI clustering groups. **(B)** the immune checkpoint relevant genes and immune-activation-relevant genes expressed in high and low ICI score subgroups. **(C,D)** The biological function of low and high ICI score groups. **(E)** Survival curve for high and low ICI score groups in the TCGA-LUAD cohort.

We used the principal component analysis to count the total score of both the ICI score A and B to obtain appropriate indicators, and this score was regarded as the summed index of each patient. We utilized the X-tile software to divide the TCGA cohort into high and low ICI scores, which was shown in Figure 3A. Here, we chose six biomarkers (CD274, CTLA4, HAVCR2, IDO1, LAG3, and PDCD1) as immune-checkpoint-relevant signatures, while eight other biomarkers (CD8A, CXCL10, CXCL9, GZMA, GZMB, IFNG, PRF1, TBX2, and TNF) were treated as immune-activity-related signatures. As shown in Figure 3B, these biomarkers are overexpressed in the high ICI group by using the Wilcoxon test. Then, we used the gene set enrichment analysis (GSEA) to explore the underlying biology functions. The GSEA results indicated that the enriched pathways in the high ICI score group was associated with various cell proliferation signaling (Figure 3C) and the low ICI score group was attributed to cell cycle pathways (Figure 3D). Meanwhile, the Kaplan-Meier plot showed that the high ICI score group had a much better survival rate than the low group (Figure 3E).

### The Correlation Between the Immune Cell Infiltration Scores and Somatic Variants

**FIGURE 4:**
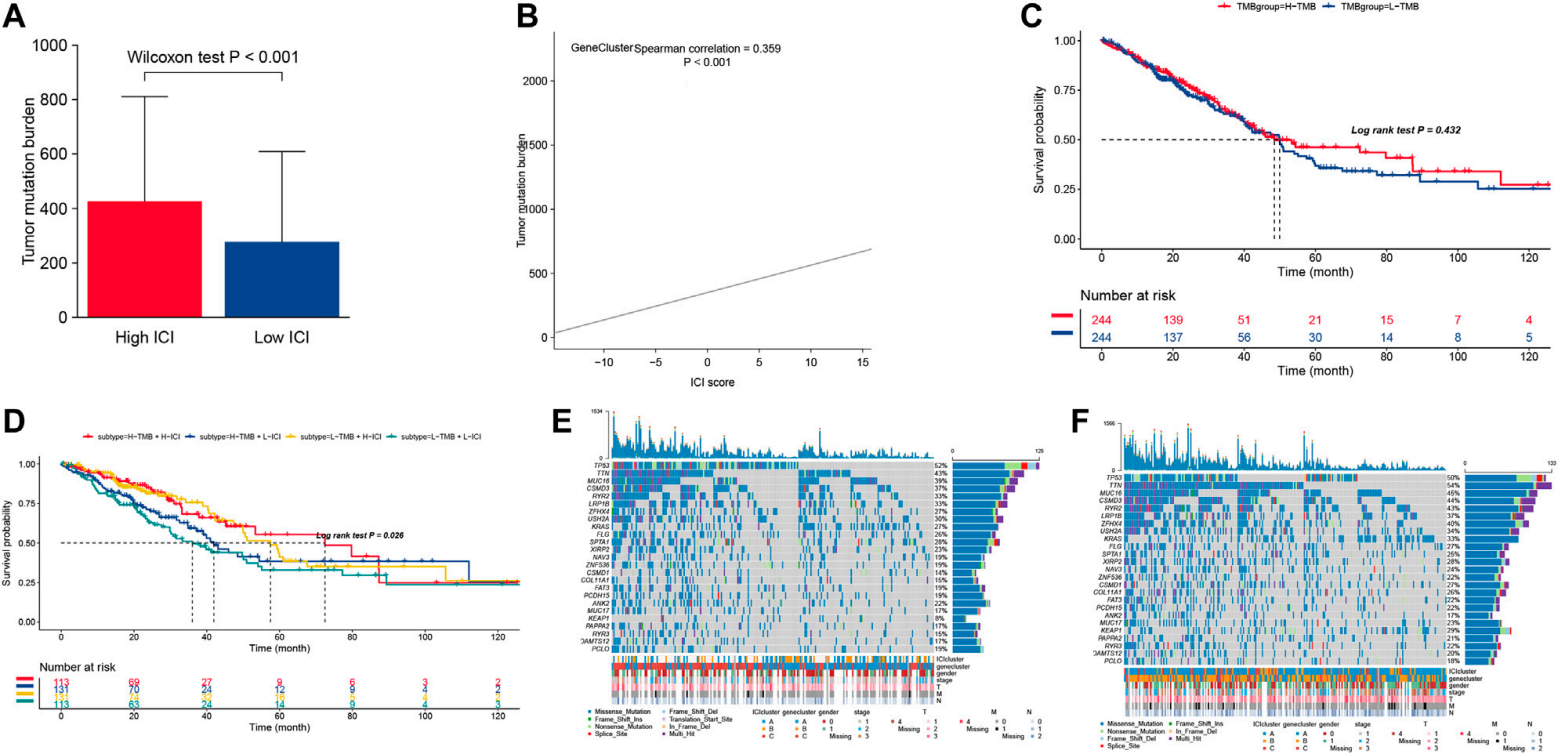
**(A)** The distribution of tumor burden mutational in different ICI score cluster. **(B)** The correlation between ICI score and TMB. **(C)** Survival curves for different TMB groups in the TCGA-LUAD cohort. **(D)** Survival curves for different subgroups stratified by both TMB and ICI scores. **(E,F)** The top 25 genes’ mutation frequency in the high ICI scores (left) **(E)** and low ICI scores on the right **(F)**.

More evidence has demonstrated that high mutation load (non-synonymous variation) in tumors were associated with increasing infiltration of CD8^+^ T cells, which showed that the tumor load mutation (TMB) may play an important role in the immunotherapy response. Based on the important role of TMB in clinical diagnosis, we aimed to study the correlation between TMB and ICI score to clarify the genetic imprinting of each ICI subgroup. Thus, we investigated the TMB between the high and low ICI scores, which showed that the high ICI score group suffered significantly higher TMB than the low ICI score group (Wilcoxon test *p* < 0.001, Figure 4A). Meanwhile, the correlation results (Figure 4B) showed that the ICI score was positively correlated with the TMB (R = 0.359, *p* < 0.001). Further study about TMB in survival was employed, and the result showed that the survival predication of high TMB was better than the low TMB group, to some extent (Figure 4C). In order to consider the association between the TMB and ICI scores, we analyzed the synergistic effect of these scores in prognostic stratification and the result showed that the overall survival of high ICI score (including ICI^high^ -TMB^high^ and ICI^high^-TMB^low^) was much better than the low ICI score (including ICI^low^ -TMB^high^ and ICI^low^-TMB^low^), and the overall survival of ICI^high^ -TMB^high^ was comparable with that of ICI^high^-TMB^low^ (Figure 4D), which indicated that high ICI score played a leading role in predicting survival. Meanwhile, the various ICI score groups in different TMB groups had significant survival differences. In conclusion, our study identified that the ICI score may be an important diagnoses index in predicted survival and evaluates the immunotherapy response.

Moreover, we assessed the driver genes’ distribution in both high ICI score and low ICI score using the MAFtools. The top 25 genes with highest frequency were pictured in both the high ICI score group and low ICI score group (Figures 4E,F), in which those genes were significantly different in both groups. These results provide one new analysis approach for studying the mechanism of tumor ICI components and gene mutations in immune therapy.

### The Role of Immune Cell Infiltration Scores in the Prediction of Immunotherapeutic Benefits

**FIGURE 5:**
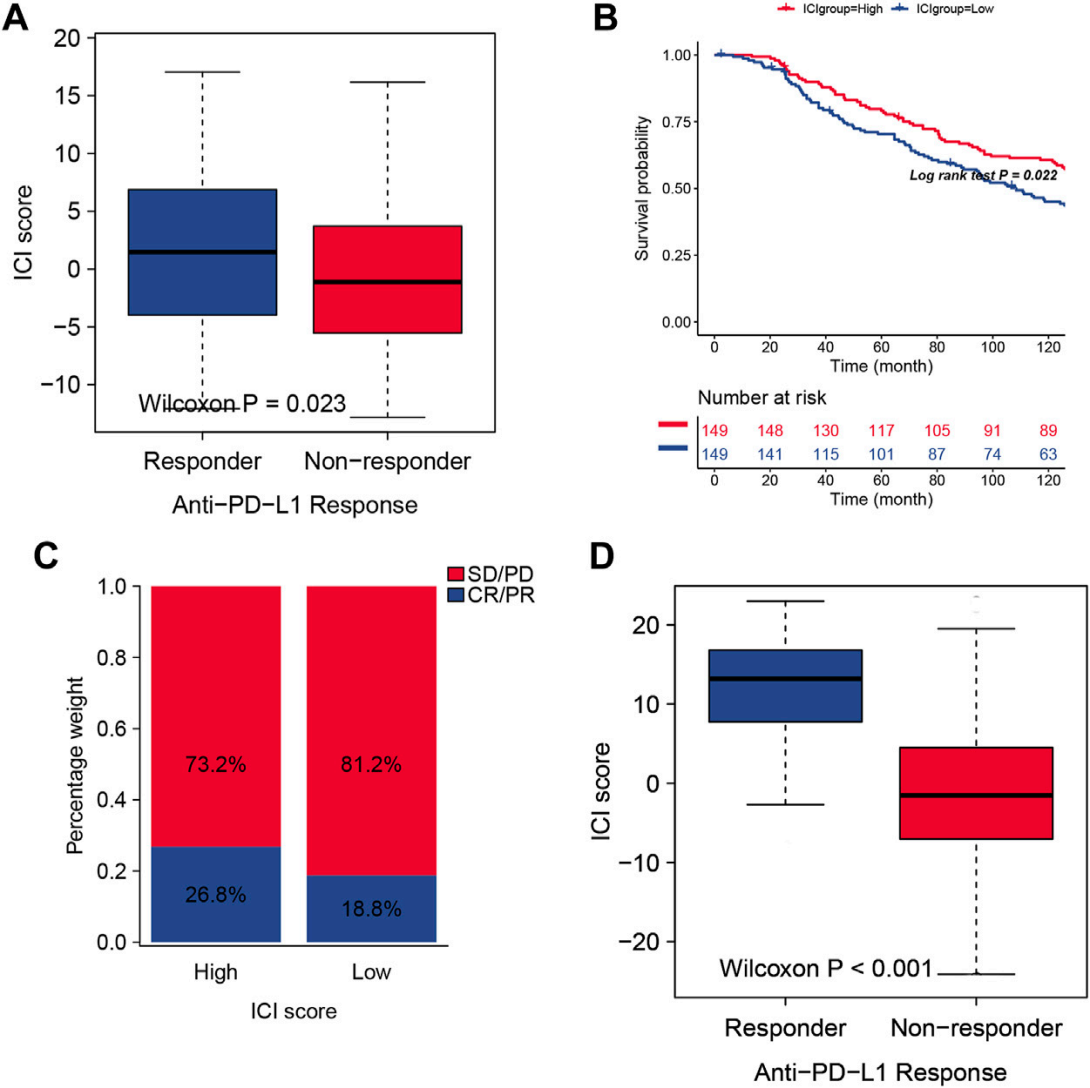
**(A)** The two ICI score groups with different anti-PD-1 response. **(B)** Survival curves for different ICI scores in the IMvigor210 cohort. 348 patients in the IMvigor210 cohort were included in the analysis. **(C)** Clinical response proportion to anti-PD-L1 immunotherapy (response/Non-response and stable disease (SD)/progressive disease (PD)) in the Imvigor210 cohort with high or low ICI groups. **(C)** The predicted Response group in TIDE analysis showed higher ICI scores than Non-response group. **(D)** The predicted Response group in TIDE analysis showed higher ICI scores than Non-response group.

Immune therapy was applied to improve the tumor therapy outcomes, especially by using PD-1 or PD-L1 specific monoclonal antibody (mAb). However, the remission rate remains lower than expected, rarely exceeding 40 percent. In order to analyze the application of ICI score in the assessment of immune therapy, we use the IMvigor210 cohort, in which these patients received the anti-PD-L1 immunotherapy, to be divided into the high ICI score group and low ICI score group. Then, we assessed the survival rates in the different ICI score groups, and the results showed that the high scoring group had much better survival than the low scoring group (Figure 5B). The objective response rate of anti-PD-L1 therapy was higher in the high ICI score group than in the low ICI group (Figure 5A). We also found that higher ICI scores are correlated with objective response to anti-PD-L1 therapy (Figure 5C).

We also performed a validation set, and we used TIDE website (Jiang et al., 2018) to predict the cancer immunotherapy response for the 535 patients from TCGA database, and our ICI score is consistent with the TIDE index from the aspect of predictive results ((Figure 5D), which can be an effective immunotherapy index for identifying suitable candidates to receive the immunotherapy. In order test whether this ICI score signature can be used as an independent prognostic factor, Cox analysis was used, and the result indicated that ICI score can be a better marker for prognosis (S2).

## Discussion

The cancer immunotherapy of PD-1/PD-L1 has significantly improved NSCLC therapy (Han et al., 2020). However, the lack of predictive biomarkers has limited its further clinical application in precise medicine (Teng et al., 2018; Camidge et al., 2019). In order to resolve these problems in immunotherapy, one method for identifying suitable LUAD patients to further receive PD-1/PD-L1 therapy should be built. In this investigation, we established a methodology to evaluate the tumor immune cell infiltration in LUAD. To our knowledge, the patient survival should be associated with the gene expression of tumor tissues (Hanahan and Weinberg, 2011). However, to simply attribute the patients to specific subtypes, e.g. oncogenic genes, did not significantly improve the therapeutic benefits. As a result, more precise attribution may be more useful for applying the patient-specific tailored therapy.

Firstly, the landscape of immune cell infiltration in LUAD was established. In this attribution, the immunological status of tumor can be well-identified. Based on the overall survival, these patients can be divided into three subtypes; patients in ICI Cluster 2 had a favorable prognosis. By analyzing the infiltration of immune cells, we found that the infiltration percentage of several immunological cells in ICI cluster 2 is higher than other groups, including Dendritic cells resting, Dendritic cells activated, Macrophages M2, Mast cells resting, NK cells activated, and T cells CD4 memory resting. Moreover, the immunoscore and stromalscore is also higher. Considering the favorable prognosis of ICI Cluster 2, these results indicate that the overall survival of.

LUAD patients may be benefitting from the higher immune cell infiltration and implied that the immunological status of these tumors is “hot”. The “hot” tumor tissue may be more sensitive for immune-therapy (Brockwell and Parker, 2019).

Then, we fetched the immune-related genes expression on the basis of aforementioned clusters. Difference expression genes (DEGs) were further utilized for characterizing the expression condition and classified as two subtypes: ICI signature genes A (144 DEGs) and B (64 DEGs). Interestingly, the gene profiles of these DEGs displayed the difference based on the classification of ICI signature. And the expression levels of ICI signature gene A in ICI gene cluster B and C displayed a higher ICI signature score and immune cell infiltration. Gene cluster A exhibits a higher ICI signature than gene B. Consistent with these findings, the patients in gene cluster A exhibit better prognosis than gene cluster B and C. The anti-cancer immune response in gene cluster A is associated with favorable prognosis and we believe that the patients in gene cluster A may benefit from immunotherapy. In order to identify the role of higher immune cell infiltration in improving patient survival, the GSEA analysis was performed, and the results showed that the immunological response-related pathways have been well activated, such as pathways related to positive regulation of cytokine production (Giles et al., 2018; Fisher et al., 2019), regulation of immune effector process (Wu et al., 2020b), and leukocyte proliferation (MollicaPoeta et al., 2019). And the relative biomarker expression of cytokines also confirmed this result. These results demonstrated that immune cell infiltration is significantly involved in immune response.

Previous studies suggested high mutation load in tumors were associated with increasing infiltration of CD8^+^ T cells. We also explored the immunological infiltration on genome stability, and tested the TMB frequency based on the ICI score. From the analysis, it was seen that the higher ICI score indicated the higher tumor mutation burden and meant a worse prognosis, while high ICI score but not high TMB played a leading role in predicted survival. A recent study claimed that high tumor mutation burden fails to predict immune checkpoint blockade response across all cancer types (McGrail et al., 2021), which supported our conclusion.

In order to identify the correlation between ICI score andimmunological response, we analysed the association between anti-PD-L1 response and ICI score. The higher ICI score of LUAD patients suffered the better responder. We found that the response rate of anti-PD-L1 therapy in the high ICI score group was much higher than the low ICI group, confirming that ICI scoring can be used as a predictive index for LUAD before receiving PD-1/PD-L1 therapy.

Recently, numerous studies have focused on predicting prognosis of lung cancer patients with immune-related genes (Song et al., 2019; Jiang et al., 2020). In 2011, Xie Y et al. developed a prognostic signature based on 100 Non–Small-Cell lung cancer (NSCLC) FFPE samples and refined a 59-gene lung cancer prognosis signature (Xie et al., 2011). This differed from the existing research which mainly focused on the expression of a few specific genes. Our study divided patients into different groups using the CIBERSORT algorithm. Then, ICI scores were established based on PCA analysis of DEG between clusters. This process reduces the inauthenticity of CIBERSORT results (Vallania et al., 2018). This scoring system proved to be more stable and may not be affected by the expression of single or multiple genes (Huang et al., 2021; Jiang et al., 2021; Xu et al., 2021). In addition, the established ICI scoring system can provide valuable information for the design of alternative treatment regiments.

There is a limitation in this study, in that this study only used public data for analysis, and did not involve *in vivo* or *in vitro* experimental verification. The experimental verification is required in the future to promote our understanding about the landscape of immune cell infiltration and tumor immune response.

In conclusion, we have analyzed the landscape of immune cell infiltration and established one approach for evaluating the immune response condition of LUAD. A higher ICI score was identified to be correlated with tumor immune response and this evaluating tool could be an ideal index to identify suitable candidates to receive immunotherapy.

## Data Availability

Publicly available datasets were analyzed in this study. This data can be found here: TCGA; GEO.
